# Continuous renal replacement therapy with cytokine-adsorbing hemofilter to control resuscitative endovascular balloon occlusion of the aorta-related ischemia-reperfusion injury in a swine hemorrhagic shock model

**DOI:** 10.1007/s00068-024-02707-4

**Published:** 2025-01-24

**Authors:** Yosuke Hayashi, Yoshimitsu Izawa, Yasutaka Tanaka, Makoto Aoki, Yosuke Matsumura

**Affiliations:** 1 Department of Emergency and Critical Care Medicine, Chiba University Graduate School of Medicine, 1-8-1 Inohana, Chiba, 260-8677 Japan; 2 Department of Emergency and Critical Care Medicine, Jichi Medical University, 3311-1 Yakushiji, Shimotsuke, 329-0498 Japan; 3 Division of Traumatology, National Defense Medical College Research Institute, 3-2 Namiki, Tokorozawa, 359-8513 Japan; 4 Chiba Emergency and Psychiatric Medical Center, Department of Intensive Care, 6-1 Toyosuna, Chiba, 261-0024 Japan

**Keywords:** Resuscitative endovascular balloon occlusion of the aorta, Hemorrhagic shock, Ischemia-reperfusion injury, Continuous renal replacement therapy, Cytokine-absorbing hemofilter

## Abstract

**Purpose:**

Resuscitative endovascular balloon occlusion of the aorta (REBOA) is beneficial for uncontrollable torso bleeding; however, prolonged REBOA causes ischemia-reperfusion injury. The purpose of this study is to examine the hypothesis that continuous renal replacement therapy (CRRT) with a cytokine-adsorbing hemofilter would improve mortality due to hemorrhagic shock with REBOA-reperfusion injury by controlling metabolic acidosis, hyperkalemia, and hypercytokinemia.

**Methods:**

Hemorrhagic shock with 40% blood loss was induced by phlebotomy in eight female swine. CRRT was performed on four swine after 90 min of REBOA, and the remaining four swine (control group) underwent the same procedures except for CRRT. We evaluated the survival time and trends of pH, HCO^3−^, potassium, lactate, circulatory inflammatory cytokines, and histopathology of the intestine for 180 min after REBOA deflation.

**Results:**

Two swine in the CRRT group and one in the control group survived; no significant difference were observed in survival rates between the groups (*p* = 0.45). Furthermore, no significant differences in the transition of biomarkers and histopathological grades were observed between the groups. The CRRT group showed a tendency of increasing pH and HCO^3−^, decreasing lactate, lower elevation of potassium and cytokine levels (interleukin 6, CRRT: 1008.5 [770.4–1246.6], control; 1636.7 [1636.7–1636.7] pg/mL at t = 270), and lower intestine histopathological grade (jejunum, CRRT; 1.5 [1.3–1.8], control; 4.0 [4.0–4.0], ileum, CRRT; 1.5 [1.3–1.8], control; 4.0 [4.0–4.0] at t = 270) than the control group.

**Conclusions:**

CRRT may mitigate acute-REBOA-related ischemia-reperfusion injury by controlling biomarkers. Further research is required to evaluate the impact on long-term mortality.

**Supplementary Information:**

The online version contains supplementary material available at 10.1007/s00068-024-02707-4.

## Introduction

Hemorrhagic shock is a major cause of death in the acute phase of trauma injury, and controlling bleeding is essential for decreasing mortality [1, 2]. Resuscitative endovascular balloon occlusion of the aorta (REBOA) is a beneficial bleeding-control device that is less invasive than aortic cross-clamping for use under emergency room thoracotomy for the resuscitation of torso hemorrhage [3–5]. However, prolonged REBOA can cause a problematic complication, which is ischemia-reperfusion injury (IRI); the resulting metabolic acidosis, hyperkalemia, and organ failure can lead to fatal conditions [6, 7].

Establishing treatment methods to control REBOA-related ischemia-reperfusion injury (R-IRI) not only improves poor prognosis caused by R-IRI but can also allow the use of REBOA to rescue more patients with uncontrollable bleeding. While various pharmacological therapies [8, 9] and targeted temperature management [10] for R-IRI have been reported, there is no established treatment strategy yet.

Some studies reported that continuous renal replacement therapy (CRRT) is effective in controlling metabolic acidosis and hyperkalemia associated with acute leg ischemia [11] and crush syndrome [12], which have similar pathophysiology to that of R-IRI; however, there are no reports on the effectiveness of CRRT for R-IRI. Notably, hypercytokinemia is considered to be a cause of cell death in IRI [13, 14], similar to that in sepsis [15]. Because CRRT with a cytokine-adsorbing hemofilter (CAH) controls hypercytokinemia [15], it may be effective in controlling hypercytokinemia in R-IRI.

We hypothesized that hemodialysis, particularly CRRT with CAH, will improve mortality due to hemorrhagic shock with R-IRI by controlling metabolic acidosis, hyperkalemia, and hypercytokinemia. In this pilot study, we performed CRRT in a swine hemorrhagic shock model after inflating REBOA for 90 min, the longest REBOA inflation in previous reports in a swine model [16], and examined trends in blood gas analysis (BGA) data. We also evaluated the intestinal histopathological damage caused by R-IRI and differences in circulatory inflammatory cytokine levels.

## Methods

### Animals

To minimize variability due to age and sex differences, we used eight (four pigs each for the CRRT and control group) 3–4-month-old female non-pregnant domestic pigs weighing 35–45 kg (Landrace: purchased from Sanesu Breeding Co., Ltd. Chiba, Japan). This study was conducted in an accredited animal research laboratory (Centre for Development of Advanced Medical Technology [CDAMTec], Jichi Medical University, Tochigi, Japan). All methods were performed in accordance with the relevant guidelines and regulations and were approved by the Institutional Animal Experiment Committee (authorization number: 21006-01). The ARRIVE guidelines (https://arriveguidelines.org) was used to ensure proper reporting of methods, results, and discussion. Due to the nature of the pilot study, power analysis was not performed. The number of experimental animals was decided as four in each group following the agreement with the Institutional Animal Experiment Committee on the large animal experiment based on the Three Rs Principles (Replacement, Reduction, and Refinement), especially from a point of Reduction.

### Animal preparation

The grouping of the animals was randomized by the technicians of CDAMTec. The researchers were not informed pre-experimental condition of the animals. Anesthesia was induced and maintained as in a previous report [17]. The animals were premedicated intramuscularly with 0.06 mg/kg medetomidine (Nippon Zenyaku Kogyo Co., Ltd., Fukushima, Japan), 0.3 mg/kg midazolam (Astellas Pharma Inc., Tokyo, Japan), and 0.08 mg/kg atropine (Mitsubishi Tanabe Pharma Corporation, Osaka, Japan) in the animal cage. Maintenance anesthesia consisting of 3% sevoflurane was induced and 1% propofol was injected intravenously as needed (Maruishi Pharmaceutical Co. Ltd, Osaka, Japan) after confirmation of sedation and endotracheal intubation in the animal operation room. The animals were mechanically ventilated sufficiently to maintain the end-tidal CO2 at 40 ± 5 mmHg with tidal volumes of 7–10 mL/kg and a respiratory rate of 10–15 breaths/min. The pigs were placed on a warming blanket at 39 °C to maintain body temperature. After general anesthesia induction, an arterial line catheter was placed into the right carotid artery for proximal pressure monitoring and blood sampling. The arterial line catheter was connected to a FloTrac transducer and monitor (EV 1000; Edwards Lifesciences, Irvine, CA, USA) to measure Stroke volume variation (SVV). A triple-lumen catheter (Power-Trialysis^®^; Becton, Dickinson and Company, New Jersey, United States) was inserted into the right external jugular vein to connect the CRRT circuit. A 10-Fr sheath was placed into the right femoral artery to insert a 7-Fr REBOA catheter (Rescue Balloon^®^; Tokai Medical Products, Aichi, Japan). The side arm of the 10-Fr sheath was used for distal pressure monitoring. Acetated Ringer’s solution was infused, targeting a SVV between 10 and 15%, and a bolus injection (500mL each) was administered when the mean blood pressure dropped to less than 30mmHg.

### Induction of hemorrhagic shock and REBOA placement

As the hemorrhagic shock model, phlebotomy was performed using a 10-Fr arterial sheath in the right femoral artery, and 30 mL/kg of blood (approximately 40% blood loss) was withdrawn exponentially for 20 min to induce class IV shock. The first half of this blood volume was removed at 2.15 mL/kg/min for 7 min and the remainder at 1.15 mL/kg/min for 13 min [18].

The REBOA catheter was placed in the thoracic aorta to maintain the balloon position in zone 1 and fixed. The balloon was then gradually inflated with close distal pressure monitoring. Total REBOA (100% occlusion) inflation was defined as the complete cessation of distal pulse pressure and was performed on both the CRRT and control groups for 90 min after hemorrhagic shock induction.

T = 0 was defined as after phlebotomy and immediately before REBOA inflation, and t = 90 was defined as immediately before REBOA deflation.

### CRRT setting

CRRT was performed using a JUN-55X-II (Toray Medical Co., Ltd., Japan) as a bedside monitoring console. CH-1.8 W (Toray Medical Co., Ltd., Japan), made of a polymethyl methacrylate membrane, was used as the CRRT hemofilter to adsorb cytokines. Circuits and columns were primed with heparinized saline before treatment. CRRT treatment was performed immediately before REBOA deflation until 3 h later under the following conditions: blood flow rates, 120 mL/min; dialysate flow rates, 2000 mL/h; replacement solution flow, 2500 mL/h; and substitution fluid rate, 500 mL/h. Heparin sodium (30 U/kg/h) was continuously administered as an anticoagulant from REBOA inflation to the end of observation. The control group was administered the same amount of heparin sodium for the same duration. The grouping was not blinded during the experimental procedure.

### Blood gas analysis and cytokine analysis

Arterial BGA was performed using a portable BGA measuring device, Epoc^®^ (Siemens Healthineers, Bayern, Germany) before phlebotomy as the baseline values and at t = 0, 90, 150, 210, and 270. Serum cytokine assay was performed at t = 0, 90, 150, 210, and 270 and commissioned with Eurofins Clinical Testing Service Japan Co. (Tokyo, Japan) using Luminex^®^ Multiplex Assays (ThermoFisher Scientific, Massachusetts, US).

### Intestinal histopathology

To collect pathological specimens of the intestine, the jejunum (10 cm from the proximal) and ileum (10 cm from the distal) were resected. The specimen sections were then stained with hematoxylin and eosin (H&E), examined by three physicians and surgeons, and classified according to a grading system of tissue damage, as described in previous studies (details are shown in **SI Methods**) [19–21]. The grouping was not blinded during the histological classification.

### Statistical analysis

Data are expressed as median (interquartile range) for continuous values. Differences between the two groups were analyzed using the Mann–Whitney U test, and survival rate analysis was performed using the Kaplan–Meier method and log-rank test. Statistical significance was set at *p* < 0.05. significant. All statistical analyses were performed using the SPSS software version 26.0.0 (IBM Corporation, Armonk, NY, USA).

## Results

### Baseline characteristics of a hemorrhagic shock model

Baseline characteristics of swine in the CCRT and control groups are shown in SI Table S1. The heights of swine in the CRRT and control groups were 106.0 (105.0–107.5) vs. 105.5 (104.8–106.3) cm (*p* = 0.49), respectively, and their weights were 37.7 (37.0–38.4) vs. 38.2 (37.2–39.0) kg (*p* = 0.89), respectively. No significant differences were observed in the total phlebotomy volume between the CRRT and control groups (1130.0 [1071.3–1180.0] vs. 1130.0 [1080.0–1180.0] mL; *p* = 0.89).

BGA results before and after phlebotomy revealed no significant differences in pH, HCO^3−^, base excess, potassium, hemoglobin, hematocrit, lactate, or creatinine in either group.

### Effect of CRRT on survival

**Fig. 1:**
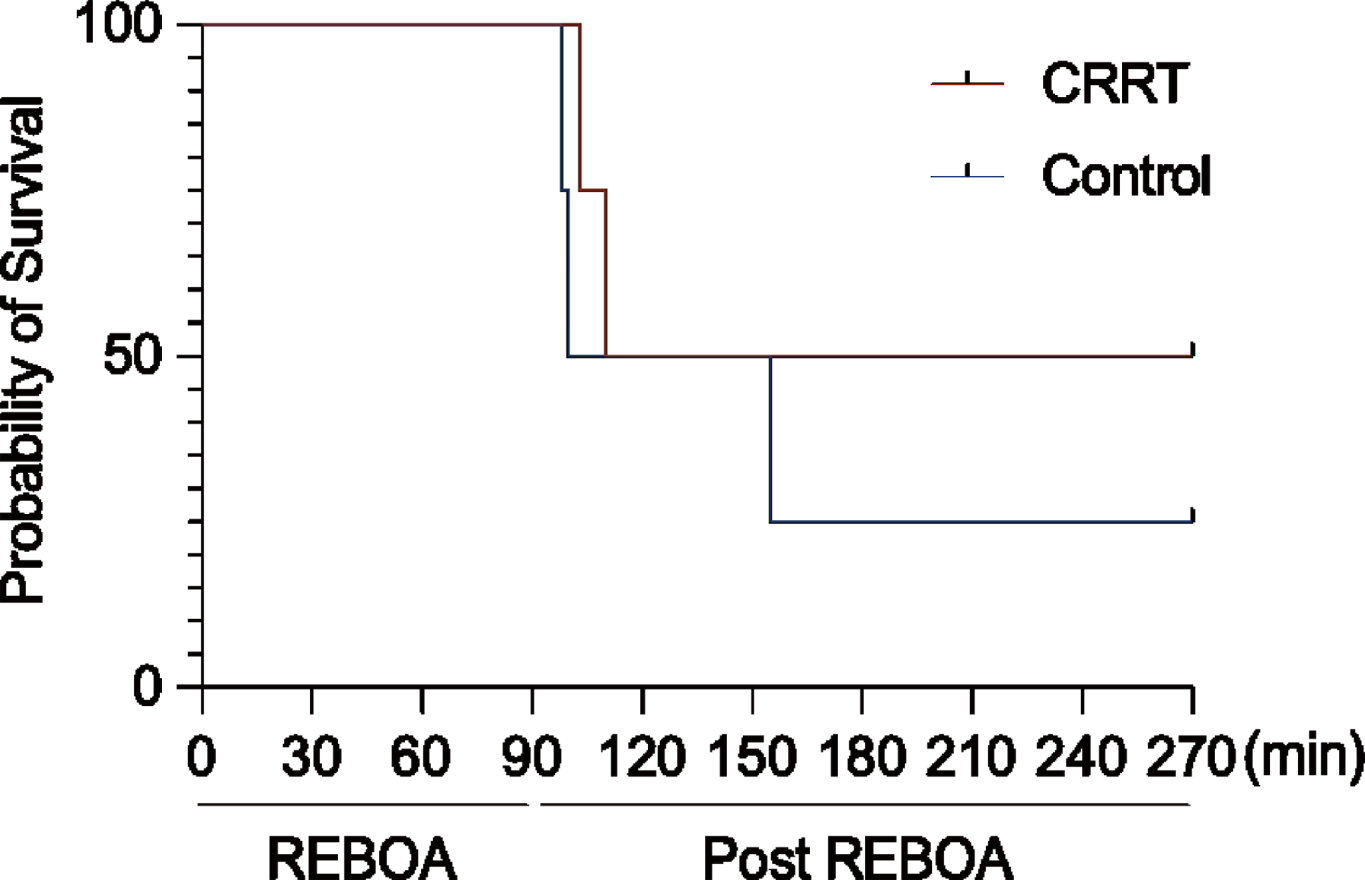
Survival analysis of swine in the CRRT and control groups. No significant difference is observed; however, the survival rates tend to be higher in the CRRT group. CRRT, continuous renal replacement therapy; REBOA, resuscitative endovascular balloon occlusion of the aorta

A comparison of survival rates between the CRRT and control groups is shown in Fig. 1. All swine survived during the hemorrhagic shock induction and REBOA phases; two swine in each group died immediately after REBOA deflation, and one in the control group died 1 h after REBOA deflation. Finally, two and one swine in the CRRT and control groups, respectively, survived; no significant differences were observed between the two groups (*p* = 0.45).

### Effects of CRRT on the measurements of arterial blood gas analysis

**Fig. 2:**
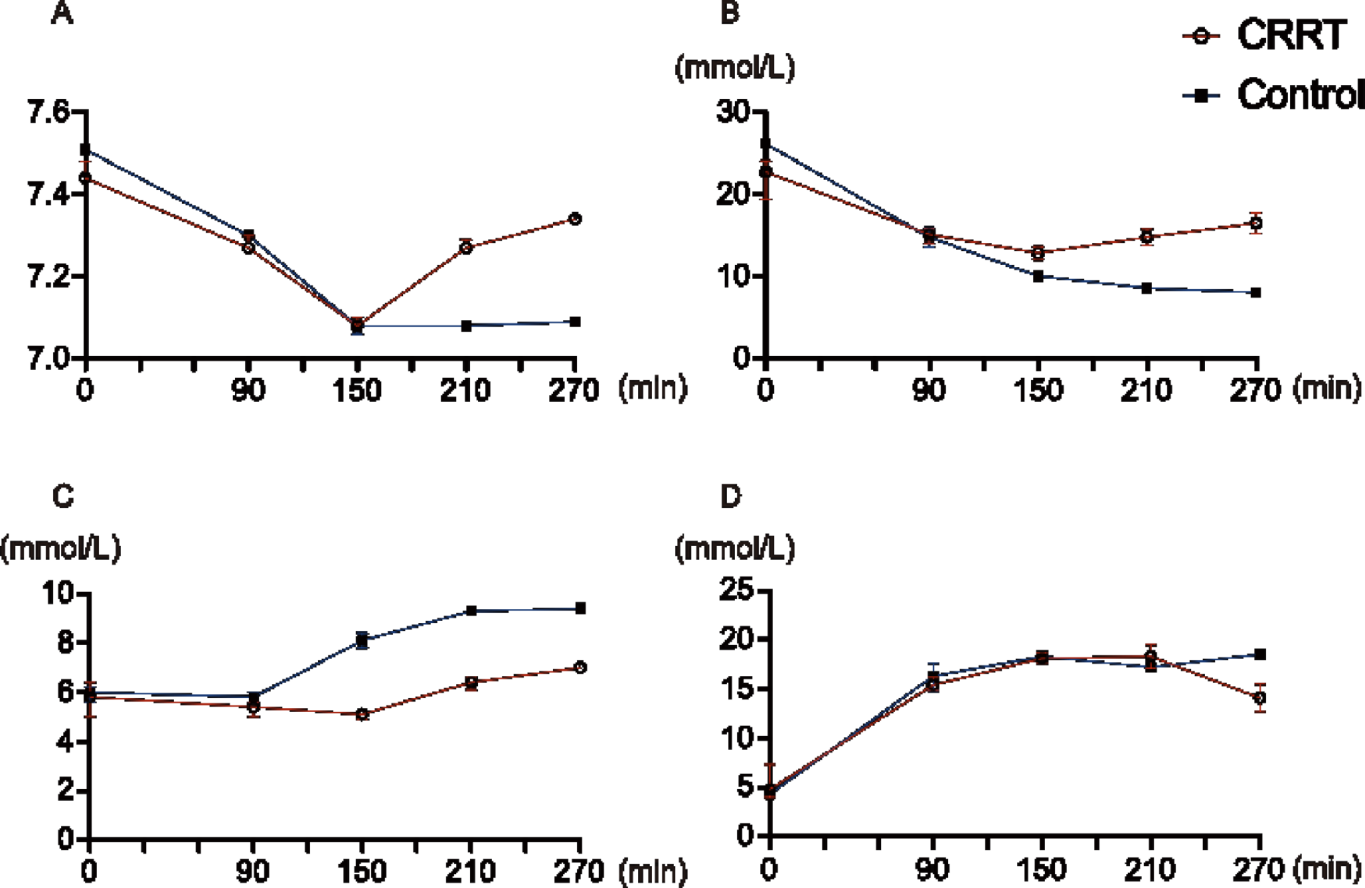
Changes in blood gas analysis in the CRRT and control groups. No significant difference is observed; however, the CRRT group shows a trend of increasing (**A**) pH and (**B**) HCO^3−^ levels, whereas the control group show low levels. (**C**) The potassium levels are lower in the CRRT group than in the control group. (**D**) Lactate levels decreases at t = 270 only in the CRRT group. Data are expressed as median and interquartile range (error bar). The number of swine is decreased because of death (*n* = 4 at t = 0 and *n* = 2 at t = 150, 210, and 270 in the CRRT group; *n* = 4 at t = 0, *n* = 2 at t = 150, and *n* = 1 at t = 210 and 270 in the control group). CRRT, continuous renal replacement therapy

The number of analyzed swine was decreased because of death (*n* = 4 at t = 0 and *n* = 2 at t = 150, 210, and 270 in the CRRT group; *n* = 4 at t = 0, *n* = 2 at t = 150, and *n* = 1 at t = 210 and 270 in the control group). No significant differences in pH, HCO^3−^, potassium, and lactate levels were observed between the CRRT and control groups, although the CRRT group showed a tendency toward increased pH and HCO^3−^, lower elevation of potassium, and decreased lactate levels (Fig. 2; Table 1).

**Table 1:** Trend of blood gas analysis results in the CRRT and control groups

	CRRT	Control	*p*-value	
pH				
t = 90	7.27 (7.24–7.30)	7.30 (7.29–7.32)	0.34	
t = 150	7.08 (7.07–7.10)	7.08 (7.06–7.10)	1.00	
t = 210	7.27 (7.26–7.29)	7.08 (7.08–7.08)	0.67	
t = 270	7.34 (7.32–7.35)	7.09 (7.09–7.09)	0.67	
HCO^3−^, mmol/L				
t = 90	15.2 (14.1–15.8)	14.8 (13.6–15.9)	1.00	
t = 150	12.9 (12.1–13.6)	10.1 (9.9–10.2)	0.33	
t = 210	14.8 (13.8–15.8)	8.6 (8.6–8.6)	0.67	
t = 270	16.5 (15.3–17.8)	8.1 (8.1–8.1)	0.67	
K^+^, mmol/L				
t = 90	5.4 (5.0–5.6)	5.8 (5.6–6.0)	0.20	
t = 150	5.1 (4.9–5.2)	8.1 (7.8–8.4)	0.33	
t = 210	6.4 (6.1–6.6)	9.3 (9.3–9.3)	0.67	
t = 270	7.0 (6.9–7.0)	9.4 (9.4–9.4)	0.67	
Lactate, mmol/L				
t = 90	15.4 (14.9–16.2)	16.3 (14.8–17.6)	0.89	
t = 150	18.1 (17.6–18.7)	19.2 (18.8–19.6)	0.67	
t = 210	18.3 (17.1–19.5)	17.2 (17.2–17.2)	1.00	
t = 270	14.1 (12.7–15.5)	18.5 (18.5–18.5)	0.67	

CRRT, continuous renal replacement therapy

Data are presented as a median and interquartile range for continuous variables; p*-*values were calculated using the Mann–Whitney U test

The number of swine is decreased due to death (*n* = 4 at 0 min and *n* = 2 at 150, 210, and 270 min in the CRRT group; *n* = 4 at 0 min, *n* = 2 at 150 min, and *n* = 1 at 210 and 270 min in the control group)

In both groups, the pH was lower at t = 90 than that at t = 0. Further, the pH decreased in both groups at t = 150, although it should be noted that only data of surviving cases were analyzed. At t = 210 and 270, the CRRT group showed an increasing trend, whereas the trend for the control group remained low (Fig. 2A). Similar to the trend for pH, HCO^3−^ tended to decrease in both groups at t = 90 and t = 150 and increase in the CRRT group only at t = 210 and t = 270 (Fig. 2B). In both groups, the potassium level was high at t = 90, and only the potassium level in the control group was further elevated at t = 150. The potassium level was elevated at t = 210 and t = 270 in both groups; however, the CRRT group exhibited a lower elevation than the control group (Fig. 2C). Lactate level was elevated at t = 90, t = 150, and t = 210 in both groups and decreased at t = 270 only in the CRRT group (Fig. 2D).

### Effects of CRRT on intestinal histopathology

**Fig. 3:**
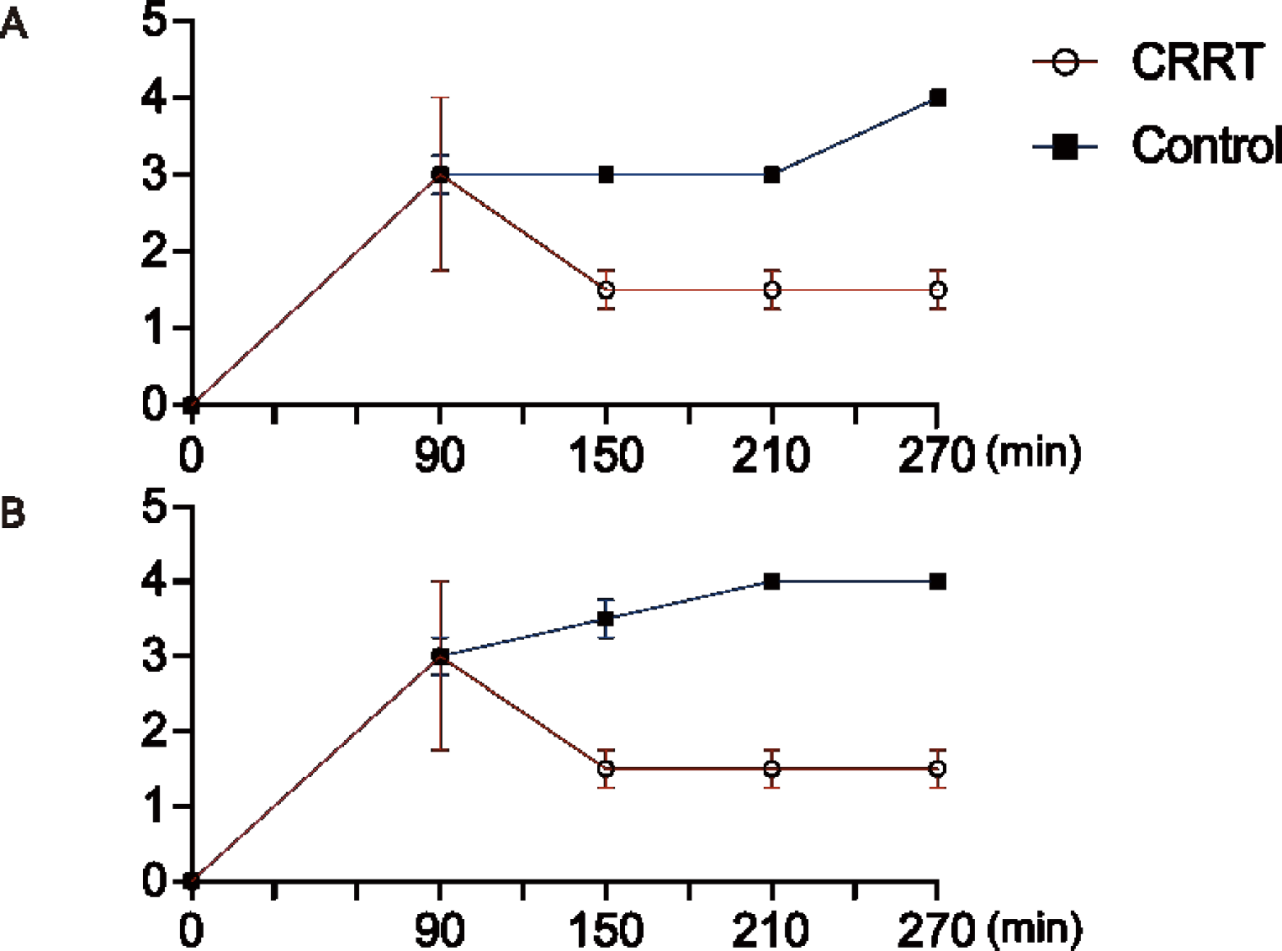
Histopathological changes in the CRRT and control groups. No significant differences are observed in the histopathological grades of the (**A**) jejunum and (**B**) ileum; however, the CRRT group tends to have a lower grade at t = 150, 210, and 270. Data are expressed as median and interquartile range (error bar). The number of swine is decreased because of death (*n* = 4 at t = 0 and *n* = 2 at t = 150, 210, and 270 in the CRRT group; *n* = 4 at t = 0, *n* = 2 at t = 150, and *n* = 1 at t = 210 and 270 in the control group). CRRT, continuous renal replacement therapy

The number of analyzed swine was decreased because of death as mentioned above. The number of analyzed was the same as described above. No significant difference in the histopathological grade of the jejunum and ileum was observed between the CRRT and control groups at t = 90, t = 150, t = 210, and t = 270; nevertheless, the CRRT group tended to have a lower grade at t = 150, 210, and 270 (Fig. 3 and SI Fig S1, S2).

### Effects of CRRT on serum cytokine levels

**Fig. 4:**
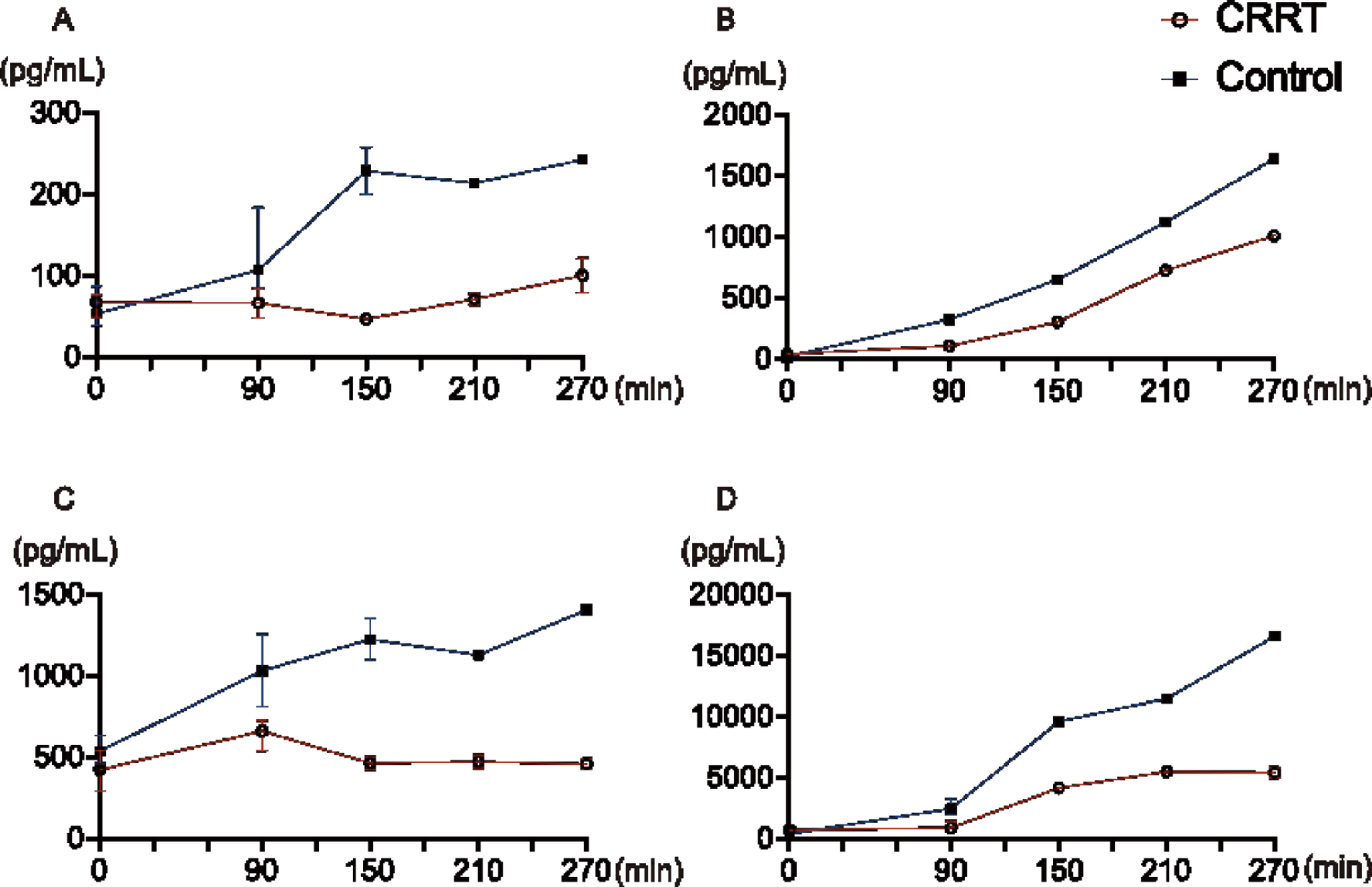
Changes of cytokine levels in the CRRT and control groups. No significant difference is observed; however, a certain trend is observed in (**A**) interleukin (IL)-1b, (**B**) IL-6, (**C**) IL-12, and (**D**) IL-18 levels. Data are expressed as median and interquartile range (error bar). The number of swine is decreased because of death (*n* = 4 at t = 0 and *n* = 2 at t = 150, 210, and 270 in the CRRT group; *n* = 4 at t = 0, *n* = 2 at t = 150, and *n* = 1 at t = 210 and 270 in the control group). CRRT, continuous renal replacement therapy between the two groups

The number of analyzed swine was decreased because of death as mentioned above. The trends in serum cytokine levels are shown in SI Table S2. No significant difference in serum cytokine levels was observed between the CRRT and control groups; however, the CRRT group showed a tendency toward lower elevation of circulatory inflammatory cytokines such as interleukin (IL)-1b, IL-6, IL-12, and IL-18 (Fig. 4 and SI Table S2).

IL-6 levels increased in both groups, although, the increase was lower in the CRRT group (Fig. 4B). IL-1b, IL-12, and IL-18 levels increased continuously in the control group but showed no increase in the CRRT group (Fig. 4A and C, and 4D).

## Discussion

This study represents one of the first attempts to examine the effectiveness of CRRT for R-IRI. We performed CRRT for 3 h in a swine hemorrhagic shock model and observed no apparent adverse events. No significant differences in the survival rate and transition of biomarkers and histopathological grades were observed between the CRRT and control groups. However, our study revealed that CRRT with CAH tended to improve metabolic acidosis, hyperkalemia, and hypercytokinemia associated with R-IRI and reduce damage to the intestine. This finding suggests that CRRT with CAH is effective in controlling R-IRI.

REBOA is an effective and less invasive aortic occlusion procedure for uncontrollable torso hemorrhage; however, recent randomized control trials and meta-analyses have not shown that REBOA resulted in any significant difference in mortality [22, 23]. Although bleeding control is the most important contributor to mortality in patients with severe trauma [1, 2], the control of subsequent complications is also important [14]. IRI is one of the most common and critical complications of REBOA, making establishing treatment strategies for IRI an important research topic [6, 7]. In the present study, survival tended to be longer in the CRRT group, although this difference was not statistically significant. Half of the swine in both groups died immediately after REBOA deflation, suggesting that overcoming this timing is the first step in improving the prognosis of R-IRI. Notably, only one swine in the control group died 1 h after REBOA deflation. Metabolic acidosis and hyperkalemia tended to improve only in the CRRT group, which may have contributed to lower mortality in this group. Although we investigated until up to 3 h after REBOA deflation in this study, this tendency of survival could be more pronounced with further observation, necessitating further research with longer observation times is needed.

In the pathophysiology of IRI, hypercytokinemia due to oxidative stress caused by mitochondrial dysfunction leads to cell death [13, 14]. A similar mechanism has been studied in sepsis, and modulating hypercytokinemia with CRRT and CAH is reported to improve patient’s outcome [15, 24]. We applied this to R-IRI and observed that CRRT with CAH modulated hypercytokinemia in R-IRI; furthermore, we demonstrated that CRRT with CAH modulated cytokines, including IL-1b, IL-6, IL-12, and IL-18. The improvement in mortality in the CRRT group may be attributable to the improvement of metabolic acidosis and hyperkinemia and the modulation of hypercytokinemia. The effect of CRRT on outcomes from this point could also be greater with longer observation period.

Various methods have been reported for controlling R-IRI. Partial REBOA minimizes distal organ ischemia by regulating the aortic flow via partial balloon inflation. Changes in distal organ blood flow following changes in balloon volume during partial REBOA have been previously reported [17, 25]. Partial REBOA has been applied clinically and is expected to improve mortality; however, its effectiveness has not been demonstrated in a recent clinical research [26]. While some studies have shown that target temperature management [10]; administration of a cocktail of adenosine, lidocaine, and magnesium [8]; and administration of elamipretide [9] were effective in reducing the R-IRI-induced damage, these approaches have yet to be applied in clinical trials. Our study demonstrates the effectiveness of CRRT as a novel treatment for R-IRI.

The strength of this study lies in the easily applicable results in clinical practice. CRRT is commonly performed in patients with acute kidney injury in the ICU and has already been clinically applied in conditions with similar a pathophysiology, such as acute leg ischemia [11] and crush syndrome [12]. Furthermore, intraoperative continuous hemofiltration has been reported in lower extremity ischemia surgery [27], cardiac surgery [28, 29], and liver transplantation [30]; hence, applying similar strategies, such as providing an extended time until damage control surgery using REBOA and deflating REBOA under CRRT intraoperatively, is feasible. Nevertheless, this study had several limitations. First, we observed a few cases of death-related interruptions, making the interpretation of some measurements difficult. The histopathological grade of the intestine at t = 90 tended to be higher than that at t = 150 in the jejunum and ileum in the CRRT group may be influenced by the poorly graded cases that died shortly thereafter. We inflated the REBOA for 90 min, the longest time for which survival has been reported in a previous swine study [16]. Observe more cases for a longer time may be possible by administering a vasopressor or bicarbonate, similar to the previous study. However, not administering these drugs allowed us to solely examine the effects of CRRT on R-IRI. Second, individual differences in the tolerance to metabolic acidosis and hyperkalemia could affect mortality. Survivors in the control group showed very low pH and very high potassium levels, suggesting that the swine had a high tolerance. Third, the experiment is not the trauma model but a hemorrhagic shock model by phlebotomy with anti-coagulation. The effect of heparin administration may become an issue when applying the results of this study to clinical settings. Anticoagulants can worsen hemorrhage during massive bleeding events. In this study, heparin was administered to prevent thrombus formation in the aorta during REBOA and CRRT circuit, which is similar but not same as the trauma coagulopathy. From the perspective of clinical applications, whether CRRT can be performed without anticoagulation should be further evaluated. Fourth, the results of small pediatric swine may not substitute for the adult human biological response. Fifth, this study may be underpowered based on the small number of experimental animals due to ethical welfare, and incomplete blinding during the experiment and histological evaluation.

## Conclusions

CRRT may mitigate acute-phase R-IRI by controlling biomarkers such as metabolic acidosis, hyperkalemia, and hypercytokinemia. Further research is required to evaluate its impact on long-term mortality. The establishment of treatment strategies for R-IRI, including CRRT with CAH, will improve the prognosis of patients with hemorrhagic shock.

## Electronic supplementary material

Below is the link to the electronic supplementary material.

Supplementary Material 1

## Data Availability

The datasets used and analyzed in the current study are available from the corresponding author upon reasonable request.
