# Supplementary material for: Continuous renal replacement therapy with cytokine-adsorbing hemofilter to control resuscitative endovascular balloon occlusion of the aorta-related ischemia-reperfusion injury in a swine hemorrhagic shock model
Source: Eur J Trauma Emerg Surg. 2025 Jan 24;51(1):66. doi: 10.1007/s00068-024-02707-4 (PMC11761798; doi:10.1007/s00068-024-02707-4)
Supplement: Supplementary file 1 — Supplementary Material 1 [file 68_2024_2707_MOESM1_ESM.pdf]

Supplemental Information

SI Methods: Histopathological grading system of tissue damage

SI Table S1. Baseline characteristics of swine in the CRRT and control groups.

SI Table S2. Trend of cytokine assay results in the CRRT and control groups

SI Fig S1. Histopathological changes of the jejunum in survivors

SI Fig S2. Histopathological changes of the ileum in survivors

SI Methods. Histopathological grading system of tissue damage

- Grade 0:
    - Normal mucosa histology.
- Grade 1:
    - Small cytological alterations in the cell structure.
    - Increased leukocyte presence and subepithelial space at the villus tips.
- Grade 2:
    - Cellular alterations with focused lesions and cell lysis.
    - Extension of the subepithelial space with moderate lifting (with the maximum of 25% extension).
- Grade 3:
    - In addition to cytological alterations, structural lesions in the intermediate extension and massive lifting (between 25% and 50%) are observed down the sides of the villi.
    - Presence of dilated capillaries and higher quantity of inflamed cells.
- Grade 4:
    - Structural destruction of the villosities; only traces of some villosities formed by inflamed cells and necrotic material were observed, with hemorrhage and basal glandular ulceration.
    - The destruction should be between 50 and 75% of the villosity extension.
- Grade 5:
    - Destruction of all the mucosa; no glandular structure can be observed, only the amorphous material laying on the sub-mucosa tissue.

- The destruction should be between 75 and 100% of the villosity extension.

SI Table S1. Baseline characteristics of swine in the CRRT and control groups.

	CRRT (n=4)	Control (n=4)	p-value
Height, cm	106.0 (105.0–107.5)	105.5 (104.8–106.3)	0.49
Weight, kg	37.7 (37.0–38.4)	38.2 (37.2–39.0)	0.89
Total bleeding, mL	1130.0 (1071.3–1180.0)	1130.0 (1080.0–1180.0)	0.89
Blood gas analysis			
Pre bleeding			
pH	7.53 (7.51–7.54)	7.52 (7.51–7.53)	0.89
HCO^{3-}, mmol/L	28.2 (27.4–29.1)	28.8 (27.9–29.4)	0.89
Base excess, mmol/L	5.5 (4.4–6.6)	5.7 (4.9–6.5)	0.69
K^+, mmol/L	4.4 (4.2–4.4)	4.3 (4.2–4.4)	0.89
Hemoglobin, g/dL	11.1 (10.8–11.5)	12.3 (11.5–13.0)	0.20
Hematocrit, %	32.5 (31.8–33.8)	36.0 (33.5–38.0)	0.20
Lactate, mmol/L	1.2 (0.9–1.8)	1.2 (1.0–1.3)	0.89
Creatinine, mg/dL	1.1 (1.0–1.3)	1.1 (0.9–1.3)	0.69
Post bleeding (t=0)			
pH	7.44 (7.38–7.48)	7.51 (7.51–7.52)	0.20
HCO^{3-}, mmol/L	22.8 (19.5–24.1)	26.2 (25.9–26.5)	0.06
Base excess, mmol/L	-0.9 (-5.8–1.9)	3.5 (2.7–3.9)	0.34
K^+, mmol/L	5.8 (5.0–6.4)	6.0 (5.6–6.2)	0.89
Hemoglobin, g/dL	7.5 (7.3–8.3)	7.5 (7.4–7.9)	0.89
Hematocrit, %	22.0 (21.3–24.3)	22.0 (22.0–23.3)	0.89
Lactate, mmol/L	4.8 (4.0–7.3)	4.3 (4.2–4.5)	1.00
Creatinine, mg/dL	1.1 (1.0–1.3)	1.1 (1.0–1.2)	0.69

CRRT, continuous renal replacement therapy

Data are presented as a median and interquartile range for continuous variables; p-values were calculated using the Mann–Whitney U test.

SI Table S2. Trend of cytokine assay results in the CRRT and control groups.

	CRRT	Control	p-value
IL-1a, pg/mL			
t=0	27.4 (17.2–33.0)	22.2 (8.6–39.3)	0.89
t=90	18.3 (10.5–26.9)	32.1 (14.1–64.1)	0.49
t=150	13.3 (8.5–18.0)	49.4 (27.9–71.0)	1.67
t=210	11.0 (7.9–14.2)	9.1 (9.1–9.1)	1.00
t=270	11.3 (9.2–13.4)	18.4 (18.4–18.4)	0.67
IL-1ra, pg/mL			
t=0	182.1 (117.6–276.9)	289.9 (225.8–320.2)	0.89
t=90	337.2 (183.5–464.6)	536.5 (365.4–826.0)	0.34
t=150	473.2 (402.9–543.4)	1641.9 (1144.8–2139.1)	0.33
t=210	1086.7 (896.2–1277.3)	1800.7 (1800.7–1800.7)	0.67
t=270	2587.5 (2110.0–3065.1)	6837.6 (6837.6–6837.6)	0.67
IL-1b, pg/mL			
t=0	67.8 (49.1–77.1)	53.6 (39.5–87.6)	1.00
t=90	67.0 (48.2–84.3)	107.7 (84.8–184.3)	0.11
t=150	47.2 (44.0–50.5)	229.6 (200.5–258.7)	0.33
t=210	71.5 (64.3–78.7)	214.3 (214.3–214.3)	0.67
t=270	101.1 (79.9–122.2)	243.1 (243.1–243.1)	0.67
IL-2, pg/mL			
t=0	116.2 (90.0–128.6)	58.7 (10.8–148.0)	0.49
t=90	86.5 (58.1–111.5)	84.8 (25.8–252.7)	1.00
t=150	42.8 (29.3–56.2)	235.0 (119.4–350.7)	1.00

t=210	37.7 (30.4–45.0)	0.2 (0.2–0.2)	0.67
t=270	26.5 (18.0–35.0)	0.0 (0.0–0.0)	0.67
IL-6, pg/mL			
t=0	39.6 (30.2–44.6)	17.4 (8.1–37.3)	0.49
t=90	105.7 (80.0–151.8)	323.5 (267.2–376.0)	0.06
t=150	302.4 (249.0–355.8)	648.6 (577.9–719.3)	0.33
t=210	723.9 (560.2–887.5)	1118.9 (1118.9–1118.9)	0.67
t=270	1008.5 (770.4–1246.6)	1636.7 (1636.7–1636.7)	0.67
IL-10, pg/mL			
t=0	294.9 (216.3–329.1)	62.8 (12.2–298.3)	0.69
t=90	221.3 (184.0–240.7)	124.5 (66.8–559.6)	0.49
t=150	113.8 (72.5–155.0)	743.8 (403.9–1083.7)	0.67
t=210	89.0 (54.7–123.3)	96.9 (96.9–96.9)	1.00
t=270	108.1 (80.6–135.6)	201.9 (201.9–201.9)	0.67
IL-12, pg/mL			
t=0	423.0 (292.5–542.8)	537.6 (463.4–636.9)	0.34
t=90	662.5 (540.3–726.3)	1031.4 (812.9–1256.1)	0.06
t=150	464.2 (425.4–503.0)	1226.7 (1096.3–1357.1)	0.33
t=210	473.6 (433.5–513.8)	1129.6 (1129.6–1129.6)	0.67
t=270	459.7 (427.0–492.3)	1407.0 (1407.0–1407.0)	0.67
IL-18, pg/mL			
t=0	711.6 (534.7–757.7)	373.4 (276.2–723.1)	0.89
t=90	880.5 (817.4–1406.4)	2464.5 (1976.7–3260.4)	0.20
t=150	4158.8 (3941.1–4376.5)	9612.6 (9446.9–9778.3)	0.33
t=210	5497.0 (5110.9–5883.1)	11486.1 (11486.1–11486.1)	0.67

t=270	5417.2 (4980.7–5853.8)	16603.6 (16603.6–16603.6)	0.67

CRRT, continuous renal replacement therapy; IL, interleukin.

Data are presented as a median and interquartile range for continuous variables; p-values were calculated using the Mann–Whitney U test.

The number of swine is decreased due to death (n = 4 at 0 min and n = 2 at 150, 210, and 270 min in the CRRT group; n = 4 at 0 min, n = 2 at 150 min, and n = 1 at 210 and 270 min in the control group).

SI Fig S1. Histopathological changes of the jejunum in survivors.

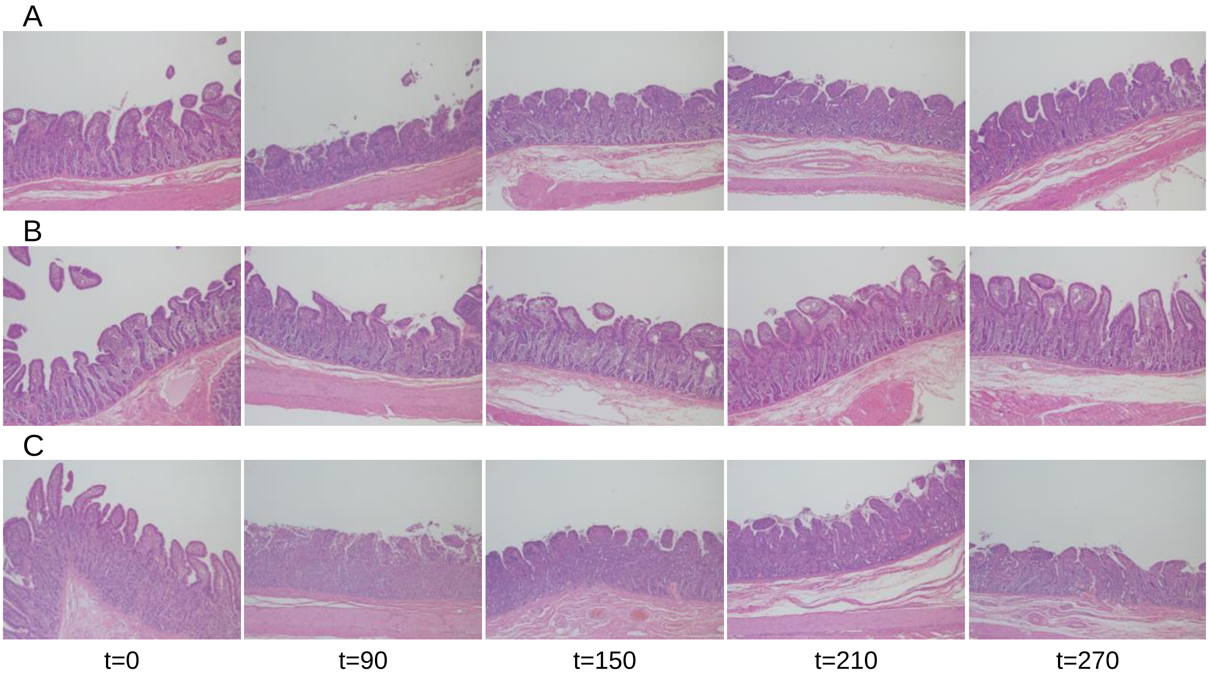

No significant difference is observed in the histopathological grade of the jejunum; however, the continuous renal replacement therapy (CRRT) group tend to have lower grades at t=150, 210, and 270.

A, B. CRRT group

A. Control group

SI Fig S2. Histopathological changes of the ileum in survivors.

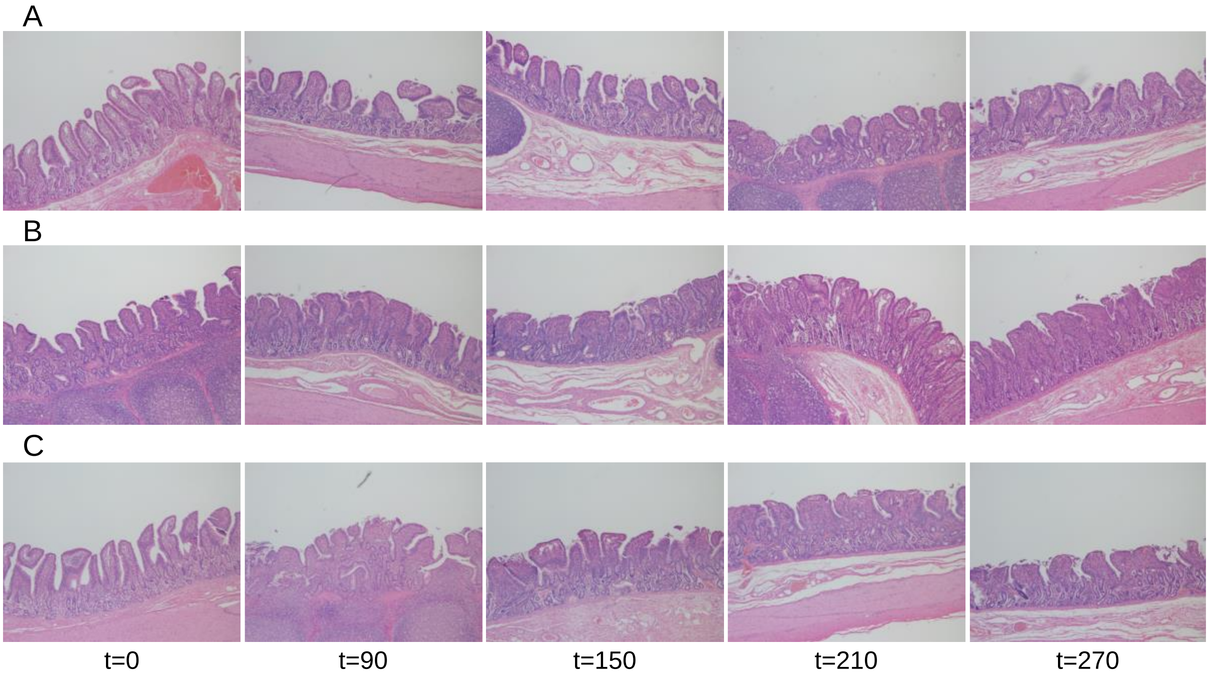

No significant difference is observed in the histopathological grade of the ileum; however, the continuous renal replacement therapy (CRRT) group tend to have lower grades at t=150, 210, and 270.

A, B. CRRT group

C. Control group
